# Enhancing Nutritional and Functional Properties of Hydroponically Grown Underutilised Leafy Greens Through Selenium Biofortification

**DOI:** 10.3390/plants14172716

**Published:** 2025-09-01

**Authors:** George P. Spyrou, Theodora Ntanasi, Ioannis Karavidas, Sofia Marka, Evangelos Giannothanasis, Lorena Vultaggio, Gholamreza Gohari, Leo Sabatino, Georgia Ntatsi

**Affiliations:** 1 Laboratory of Vegetable Production, Department of Crop Science, Agricultural University of Athens, Iera Odos 75, 11855 Athens, Greece; gspyrou@aua.gr (G.P.S.); ntanasi@aua.gr (T.N.); karavidas@aua.gr (I.K.); giannothanasis@aua.gr (E.G.); 2 Laboratory of Molecular Biology, Department of Biotechnology, Agricultural University of Athens, 11855 Athens, Greece; smarka@aua.gr; 3 Dipartimento Scienze Agrarie, Alimentari e Forestali, University of Palermo viale delle Scienze, Ed. 5, 90128 Palermo, Italy; lorena.vultaggio@unipa.it (L.V.); leo.sabatino@unipa.it (L.S.); 4 Department of Horticultural Science, Faculty of Agriculture, University of Maragheh, Maragheh 83111-55181, Iran; gohari.gh@maragheh.ac.ir

**Keywords:** malnutrition, biofortification, selenium dioxide, soilless culture, wild leafy greens

## Abstract

Nutrient intake is vital for human health, yet micronutrient deficiencies remain widespread despite sufficient calorie consumption. Biofortification is the process by which the nutrient density of food crops is increased through various strategies without altering key agronomic characteristics. This approach is widely recognised as a cost-effective method for addressing micronutrient malnutrition. When combined with the nutritional properties and inherent resilience of underutilised crops to harsh conditions, biofortification emerges as highly promising and sustainable solution. This study investigates the effects of selenium biofortification by adding different doses of SeO_2_ (0, 1, 2, and 4 μM) in the nutrient solution in three underutilised leafy vegetables [*Portulaca oleracea* L. (purslane), *Taraxacum officinale* L. (dandelion), and *Mesembryanthemum crystallinum* L. (iceplant)] grown in an open soilless system. The addition of SeO_2_ to the nutrient solution increased yield in all three species, although iceplant exhibited reduced yield at the highest SeO_2_ dose. In particular, the total yield of purslane was enhanced by 14–19% when treated with 1, 2, and 4 doses of SeO_2_, whilst the dandelion yield increased by 25% under 4 μM SeO_2_. Furthermore, the yield of iceplant increased by 14.7–17.8% at 1 and 2 μM SeO_2_. SeO_2_ application led to a dose-dependent increase in selenium concentration in the shoot tissues while remaining within safe intake limits. More specifically, selenium concentration in purslane, dandelion, and iceplant tissues increased by 92%, 91%, and 89%, respectively, at the highest SeO_2_ dose (4 μΜ) compared to untreated plants. Selenium treatment also influenced the nutritional profile of the examined plant species. With regard to the antioxidant activity, the highest recorded value was observed at 1 μM SeO_2_ for purslane and iceplant, and at 4 μM SeO_2_ for dandelion. These values were enhanced by 20%, 12%, and 27%, respectively, in comparison with 0 μM SeO_2_. In conclusion, rootzone SeO_2_ supplementation via a nutrient solution can be considered an effective biofortification strategy that enhances growth characteristics and antioxidant properties of the three investigated underutilised leafy vegetables without compromising their nutritional value.

## 1. Introduction

Access to safe and nutritious food is essential for human survival, making food safety and resilience of food supply chains critical global challenges. However, population growth, climate change, and increased urbanization are some of the key factors influencing the effectiveness and implementation of interventions aimed at addressing these challenges [1]. According to United Nations projections, the global population is expected to reach 11 billion by 2100 [2], intensifying pressure on food systems. Additionally, studies indicate that rising food demand has already led to the expansion of cropland over the past two decades [3], with estimates suggesting an additional 18% increase by 2050 [4]. This increase is attributed to the significant shrinkage of forestland and grassland areas, contributing to biodiversity loss and terrestrial ecosystem degradation [3,5,6]. In contrast, Gao et al. [7] projected a 12.8% decline of global cropland by the end of the 21st century, whilst considering the objective of maintaining the temperature increase to 1.5 °C above pre-industrial levels, as outlined in the Paris Agreement. These contrasting projections highlight the urgent need for integrated land use strategies that reconcile food production, biodiversity conservation, and climate mitigation.

Recent data from the Food and Agriculture Organization (FAO) reveal that over 713 million people currently suffer from undernutrition, with an alarming additional 152 million individuals potentially facing hunger between 2019 and 2023 [8]. Africa has been identified as the most affected region; projections suggest 582 million people could experience chronic undernourished by 2030, with the majority of these individuals residing in African nations [8]. While most prevalent in low-income countries, deficiencies in key vitamins (A and D) and minerals (zinc and iron) have also been observed in high-income nations [9,10]. Indeed, a key dimension of the global food crisis is micronutrient malnutrition, commonly referred to as “hidden hunger”, which extends beyond caloric deficiency, affecting over two billion people worldwide [11,12,13]. Critical micronutrients such as iron (Fe), zinc (Zn), iodine (I), and selenium (Se) are often absent from the daily diet, a phenomenon that can be attributed to soil nutrient depletion due to degradation. This depletion not only undermines crop nutritional quality but also poses a significant threat to global food security and nutrition [14,15]. In response, global initiatives such as the Sustainable Development Goals (SDGs), particularly Zero Hunger, and the Voluntary Guidelines on Food Systems and Nutrition (VGFSyN) [16,17] have been implemented to address these challenges.

Dietary diversification, nutrient supplementation, and food fortification are fundamental strategies for combating nutrient deficiencies. However, biofortification—enhancing crop nutrient content through breeding, genetic engineering, or agronomic practices—has been identified as the most sustainable and cost-effective solution [18,19,20,21]. Biofortification strategies are being pursued to enhance the nutrient content of high demand crops with preferred characteristics, such as high yield, focusing on malnourished rural populations who have limited access to fortified foods and supplements [22]. Unlike, conventional fortification, which involves the addition of fortificants during processing, biofortification enriches crops during cultivation [21,23]. Among biofortification strategies, agronomic biofortification—using nutrient-enhanced fertilizers—offers an effective and immediate strategy for enhancing crop nutrient content. Further optimization through advanced fertilizer formulations could maximize its potential in addressing global malnutrition [24,25].

Se constitutes an essential trace element for human health, playing a pivotal role in numerous biological functions within the human body. It contributes to cardiovascular, immune, and endocrine system functions and exhibits antioxidant properties linked to cancer prevention [26,27,28]. Se is the structural component of selenoproteins, which contain the amino acid selenocysteine and significantly influence biological activity in humans [28,29]. Both prolonged deficiency and excess Se intake can have detrimental effects, with extreme toxicity (selenosis) reported in cases of overconsumption [28]. To ensure adequate Se intake, the European Food Safety Authority (EFSA) has established age-specific recommendations: 70 μg day^−1^ for adults, 15 μg day^−1^ for infants aged 7–11 months, and 85 μg day^−1^ for lactating women, to compensate for Se secreted in breast milk [30]. The upper safe intake limit is set at 400 μg day^−1^ by multiple regulatory bodies [31,32,33].

Beyond human nutrition, Se has been studied for its effects on plant performance. According to [34] sodium selenate (Na_2_SeO_4_) application in the NS of a floating system enhanced Se accumulation in chicory and lettuce, improving yield and postharvest life. Additionally, Se application at an optimal concentration can mitigate abiotic stresses (e.g., drought and salinity) in vegetable crops [35,36]. Nevertheless, excessive Se can be phytotoxic, negatively impacting plant growth [37,38]. Plants cannot absorb SeO_2_ directly via their rootzone, and consequently it must be first converted into bioavailable forms such as SeO_4_^2−^ or SeO_3_^2−^ [39]. One possible mechanism for the indirect accumulation of SeO_2_ is the reaction of SeO_2_ with water to form H_2_SeO_3_, which is a form of Se that is available to plants [40]. Selenate is more soluble, less phytotoxic, and is absorbed more easily by plants compared to selenite [41], while the toxicity of SeO_2_ is influenced by its conversion to selenious acid. Consequently, selecting the optimal dose and species are critical for successful biofortification.

Neglected and underutilised species (NUS), are defined as wild, native crops that are not widely cultivated for commercial purposes and remain unknown to most consumers [42,43]. They are recognised for their nutritional value, resilience under harsh environmental conditions, and low crop input requirements [44,45,46,47,48,49,50]. NUS can be integrated into local, small-scale cultivation systems, enhancing dietary diversity and sustainability. Their role in addressing food insecurity and biodiversity loss has garnered significant research attention [42,51,52,53,54]. In alignment with Sustainable Development Goal 2 (SDG2), Zero Hunger, FAO launched the Future Smart Food Initiative, which seeks to improve food system sustainability and equity in hunger-prone regions [55,56]. The growing demand for nutritious and healthy foods has led to a significant increase in the consumption of NUS in both rural and urban areas [57].

Despite the heightened level of interest from researchers in the biofortification approach, an essential research gap remains with regard to the evaluation of biofortification strategies for neglected and underutilised leafy vegetables. Although these species have considerable potential and could be included in biofortification protocols due to their physiological attributes, they have not yet been thoroughly investigated. Therefore, the present study aims to examine the potential role of Se biofortification in the performance, nutritional value, and antioxidant properties of three underutilised leafy vegetables ordinarily encountered in the MED region: *Portulaca oleracea* L., *Taraxacum officinale* L., and *Mesembryanthemum crystallinum* L., commonly known as purslane, dandelion, and iceplant, respectively. Purslane is a globally distributed nutrient-rich wild leafy vegetable that is consumed in countries across Europe, Africa, the Middle East, Asia, Australia, and the Americas [58]. It contains essential bioactive compounds and it is also a valuable source of omega-3 fatty acids [58] making it a nutrient-rich food that should be included in the human diet [58]. Iceplant is a succulent known for its antioxidant and anti-inflammatory effects, attributed to its rich content of vitamins, minerals, and antioxidant compounds [59]. Iceplant is usually grown in dry regions [60] and it is extensively used as a food for its nutritional properties in several areas, such as India, Australia, New Zealand, and some EU countries [61]. Dandelion is a species of significant nutritional value that can thrive in a range of climatic conditions and is found in several regions [62]. Dandelion has been used traditionally in diets due to its high nutrient content and antioxidant properties, while several studies have focused on its additional pharmaceutical effects [63]. Finally, this study will ascertain the optimal Se dosage in relation to plant performance while considering recommended dietary intake levels. Our findings will advance understanding of Se biofortification in soilless cultivation systems, supporting sustainable agriculture and improved nutrition, particularly in Se-deficient regions (China, New Zealand, and northern Europe) [64].

## 2. Results

### 2.1. Yield and Growth Parameters

As shown in Table 1, Se enrichment in the NS significantly influenced the growth characteristics of the three plants studied. More specifically, purslane demonstrated a notable increase in fresh weight (yield), leaf number, and leaf area by approximately 14–19%, 15–30%, and 15–24%, respectively, with the incorporation of SeO_2_ in the NS. No differences among the different Se dosages were recorded. The highest SeO_2_ dose (4 μM) slightly increased plants’ dry matter content but had no significant effect on purslane’s overall growth performance. In contrast, dandelion exhibited the highest yield at 4μM SeO_2_, with increases of 25%, 16.4%, and 33.1% for fresh weight, leaf number, and leaf area, respectively. However, dry matter content decreased by 6.7% at this concentration, compared to 0 μM SeO_2_. Conversely, the highest SeO_2_ concentration in the NS adversely affected iceplant’s growth traits. Intermediate Se levels (1 and 2 μM) enhanced the fresh weight, number of leaves, and leaf area by 14.7–17.8%, 16.7–21.7%, and 23.7–24.7%, respectively. Dry matter content was highest at both 0 and 4 μM SeO_2_.

### 2.2. Plant Nutrient Profile

SeO_2_ biofortification significantly altered the accumulation of vital macro- and microelements in the three cultivated wild leafy greens. No significant changes in macroelements were observed in purslane across SeO_2_ doses (Table 2). In contrast, dandelion exhibited an approximately 8.7% increase in total nitrogen (TN) and a 34.4% increase in nitrate–nitrogen (NO_3_-N) at a SeO_2_ dose of 4 μM, while P accumulation in aboveground biomass decreased by 22.4% at the same SeO_2_ dose. The concentrations of other macronutrients (K, Ca, Mg, and Na) remained unaffected by Se treatments. In iceplant, SeO_2_ application reduced total nitrogen and NO_3_-N levels. Additionally, P, Ca, and Mg concentrations decreased at 2 and 4 μM SeO_2_. Potassium (K) levels remained stable, whereas Na increased by 25% only at the highest SeO_2_ dose.

Purslane exhibited altered micronutrient concentration after the application of SeO_2_ (Table 3). At 2 μM SeO_2_, Zn and Cu accumulation increased by 22% and 48.5%, respectively, whereas at 4 μM SeO_2_, both Zn and Cu concentrations declined. No significant differences were observed for B, Fe, and Mn among the treatments. In dandelion, SeO_2_ application significantly reduced Β concentration but increased Cu by approximately 27–28% at 2 and 4 μM SeO_2_. No significant effects were observed on Fe, Zn, and Mn levels. Iceplant’s Mn and Zn concentrations were reduced by incorporating SeO_2_ into the NS, whilst B achieved the highest concentration at 2 μM SeO_2_. Fe and Cu concentrations remained unaffected by Se application.

**Figure 1:**
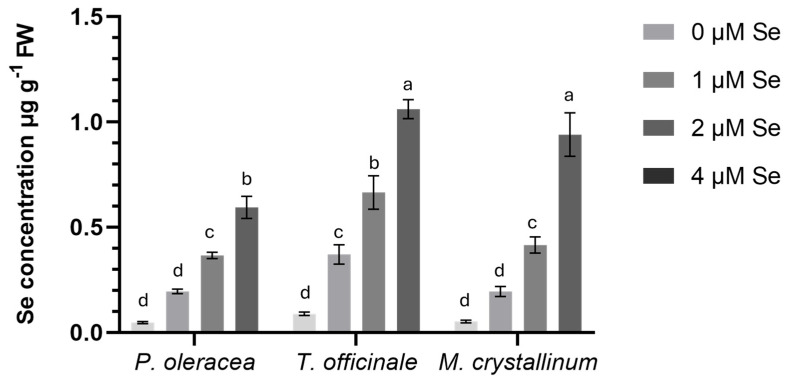
Impacts of different SeO_2_ doses and plant species on Se concentration in fresh plant tissues of the examined NUS. Each column represents the mean values of a dataset, and error bars extending above and below the column represent the standard error of the mean (SE). Different letters indicate statistically significant differences according to Duncan’s multiple range test at *p* < 0.05.

Increasing SeO_2_ concentrations in the nutrient solution (NS) progressively elevated selenium (Se) levels in the fresh biomass of all three species (Table 4). At 4 μM SeO_2_, the Se concentration in purslane increased by 92% compared to the control (0 μM). Similar trends were observed in dandelion (+91%) and iceplant (+89%). Figure 1 represents the analysis of the interaction between Se treatments and different plant species. In particular, dandelion and iceplant accumulated the highest Se levels in their edible parts (1.1 and 0.9 μg g^−1^, respectively) under 4 μM SeO_2_. In contrast, purslane exhibited the lowest Se concentration in its upper part, with concentrations 45% and 33% lower than those observed in dandelion and iceplant, respectively, at the highest dose of SeO_2_.

### 2.3. Se Intake Indices Through Biofortified NUS

As shown in Table 5, biofortified NUS are capable of meeting the recommended dietary requirements for Se when consumed as part of a balanced diet. With a daily intake of 50 g of biofortified NUS, the estimated Se intake is higher in plants treated with 4 μM SeO_2_, reaching 53 g, 47 g, and 29.7 g for dandelion, iceplant, and purslane, respectively. It is noteworthy that these values are all below the recommended upper limits. The percentage of the sufficient daily Se intake provided by NUS treated with the highest dose of SeO_2_ is 75.8% for dandelion, 67.1% for iceplant, and 42.4% for purslane. Additionally, the health risk index (HRI) was found to be minimal for all the tested plants, even at the maximum SeO_2_ dosage. These results indicate that the consumption of biofortified NUS in reasonable portions could potentially contribute to fulfilling human Se requirements.

### 2.4. Plant Biochemical Profiles

**Figure 2:**
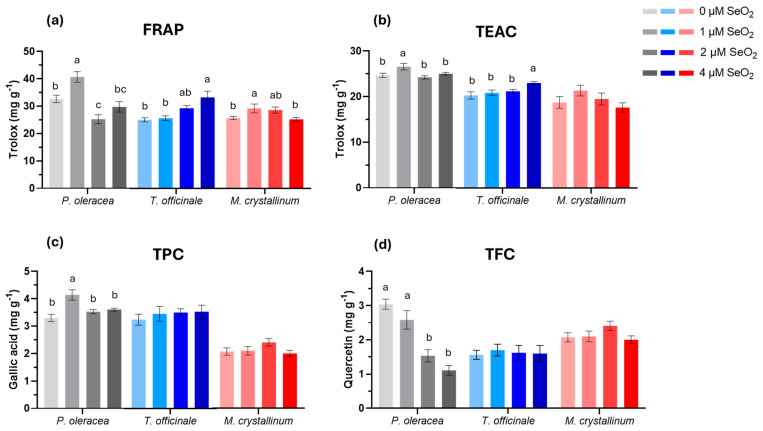
Impacts of different SeO_2_ doses on plants’ antioxidant activity: FRAP, TEAC, total phenolic (TPC), and flavonoid content (TFC). (**a**) FRAP, (**b**) TEAC, (**c**) total phenolic (TPC), and (**d**) flavonoid content (TFC). Each column on the chart represents the mean values of a dataset, and error bars extending above and below the point represent the standard error of the mean (SE). Different letters indicate statistically significant differences according to Duncan’s multiple range test at *p* < 0.05. No letter presence indicates non-significance.

The Ferric Reducing Antioxidant Power (FRAP) and Trolox Equivalent Antioxidant Capacity (TEAC) assays were used to assess the overall antioxidant capacities of purslane, dandelion, and iceplant plants. This capacity reflects the ability of plant extracts to neutralise harmful free radicals and prevent oxidative damage. As shown in Figure 2, the presence of SeO_2_ in the NS enhanced purslane’s antioxidant activity, as evidenced by both assays. In particular, purslane and iceplant plant treated with 1 μM SeO_2_ exhibited higher antioxidant performance, whereas dandelion showed improved antioxidant capacity at 4 μM SeO_2_. Furthermore, Se affected only purslane’s total phenolic and flavonoid content. The concentration of 1 μM SeO_2_ enhanced the total phenolic content by 20%, while higher Se concentrations in the NS reduced total phenolic content. In contrast, applying 2 and 4 μM SeO_2_ resulted in a 41% and 56% reduction, respectively, in TFC in purslane tissues, in comparison to the control group that received 0 μM SeO_2_. However, 1 μM SeO_2_ was able to maintain the flavonoid content of purslane plants at the same level as the control. No significant differences were observed in iceplant’s and dandelion’s TPC and TFC content under different SeO_2_ treatments.

## 3. Discussion

This study demonstrates that moderate SeO_2_ supplementation (1–2 μM) enhances growth in all three neglected and underutilised crops, whereas a high dose (4 μM) induces mild stress in iceplant. Numerous studies suggest a positive correlation between optimal Se concentrations and improved crop physiological characteristics, whether Se is applied exogenously via foliar spray, through the nutrient solution, or directly into the soil [34,65,66,67]. However, at elevated concentrations, the outcomes are often contradictory, including reduced growth, yield decline, and phytotoxicity [38,68,69]. No significant yield differences across Se treatments (0 μM, 2.6 μM, 3.9 μM and 5.2 μM) in lettuce, wild rocket, spinach, and purslane were observed by [70]. The upper tolerable Se limits for plants depend on their accumulation capacity and tissue tolerance, with species-specific variation. Consequently, plants have been classified according to this ability as hyperaccumulators (>1000 mg Se kg^−1^ DW), secondary accumulators (100–1000 mg Se kg^−1^ DW), and non-accumulators (<100 mg Se kg^−1^ DW) [71]. Although the three NUS we examined are known for their potential to accumulate diverse metals, none of them have been qualified as Se hyperaccumulators or secondary accumulators. The only detrimental effect observed was on iceplant at 4 μM SeO_2_, which exhibited altered growth traits. The findings of our study suggest that SeO_2_ biofortification at 1, 2, and 4 μM in the NS positively impacted growth performance and nutritional value in the three species. In contrast, 1 μM and 4 μM SeO_2_ were most effective in enhancing antioxidant activity, although the higher dose (4 μM) may stress certain species.

The effectiveness of biofortification depends on two primary factors: the form of Se employed, (organic or inorganic), and the biofortification method used (foliar, through NS, or soil application). Since plants do not absorb Se directly in the form of SeO_2_, it must first be converted into bioavailable forms such as SeO_4_^2−^ or SeO_3_^2−^ [39] for root uptake. Consequently, SeO_2_ has not been widely adopted in biofortification strategies. However, ref. [72] demonstrated that SeO_2_ at 2.0 μM enhanced total yield in both ungrafted and grafted cherry tomatoes grown in a soilless system. Similarly, in the current study, SeO_2_ application via the rootzone improved growth characteristics of the examined species. More specifically, purslane’s fresh biomass, number of leaves, and leaf area increased uniformly at 1, 2, and 4 μM SeO_2_. Dandelion’s fresh weight increased only at the 4 μM SeO_2_ dose, while the number of leaves and the leaf area showed an increase at 2 and 4 μM SeO_2_. Iceplant’s growth traits improved at 1 and 2 μM SeO_2_ treatments, while the highest SeO_2_ dose suppressed growth. These outcomes align with [73] who reported that foliar Na_2_SeO_4_ (260 μM) significantly increased biomass, stem length, number of stems, and diameter in wild *P. oleracea* compared to the cultivated Makovey variety, underlining the ability of Se to act as a growth stimulator at specific concentrations. Reference [68] observed similar dose-dependent effects in hydroponically cultivated *Valerianella locusta* L. treated with four distinct doses of Se using Na_2_SeO_4_ via the NS solution (0, 5, 10, 10, and 20 μM). The study concluded that plants exposed to a 5 μM Se dose increased their fresh and dry weight when harvested at 66 days. In contrast, 20 μM Se resulted in reduced growth, highlighting the narrow window between beneficial and toxic Se levels. The positive impact of Se on plants’ growth performance can be attributed to the improvement of a plant’s photosynthetic capacity and root growth, as well as improved nutritional status [35,74]. Se’s toxic effects are often attributed to malformed selenoproteins, which arise from the incorrect incorporation of Se-containing amino acids (selenocysteine or selenomethionine) in place of cysteine or methionine [75]. This misincorporation can lead to Se toxicity, disrupting cellular processes. Additionally, Se may act as a pro-oxidant, contributing to oxidative stress, despite its well-known antioxidant properties [76,77]. Studies have also linked Se toxicity with lipid peroxidation and cell membranes damage [78]. Increased lipid peroxidation in lettuce plants has been observed under high Se concentrations, particularly in plants treated with Na_2_SeO_3_ [78]. This outcome is supported by the strong negative correlation between Se leaf content and MDA content. Similar results were obtained by [79], who reported that the high Se toxicity in broccoli plants with low S nutrition was attributed to an increased Se/total Se ratio in proteins, which enhanced the generation of ROS and elevated lipid peroxidation. This caused increased cell membrane damage and reduced antioxidant enzyme activities. As shown in Table 1, purslane’s dry matter content (DMC), a common stress indicator related to growth rate [80,81], was not significantly altered under SeO_2_ treatments. On the contrary, dandelion’s DMC decreased significantly with increasing SeO_2_ doses, suggesting a potential role of Se in modulating antioxidant responses. The results of iceplant’s growth traits are in line with the observed increase in DMC in both the 0 and 4 μM SeO_2_ treatments, which may reflect mild stress under high Se exposure.

The effect of Se on macronutrient accumulation remains a contentious research topic due to its dependence on multiple factors, including plant species, Se chemical form, environmental conditions, and biofortification methods. The findings of the present study indicate that the nutritional status of dandelion and iceplant with regard to macronutrient concentration was affected by varying SeO_2_ levels. Conversely, the concentration of macronutrients in the above-ground biomass of purslane remained unaffected. More specifically, in iceplant, the total nitrogen and NO_3_^−^ content of the above-ground parts decreased under SeO_2_ exposure. In contrast, dandelion exhibited a significant increase in NO_3_^−^ concentration at 2 and 4 μM SeO_2_ and in total N at 4 μM SeO_2_. The P content in both species (iceplant and dandelion) reduced at 2 and 4 μM SeO_2_, an outcome that could be attributed to the fact that Se, mostly in the form of selenite, shares the same transport channel with P in plants [82,83]. The impacts of Se on Ca and Mg concentrations were observed exclusively in iceplant, where a decline was mainly exhibited at 4 μM SeO_2_, while Na increased at this Se level. In the research conducted by [84] mature lettuce and basil plants were treated with different concentrations of Na_2_SeO_4_ (0, 1, and 3 mg Se L^−1^ for lettuce and 0, 2, and 3 mg Se L^−1^ for basil) prior to transplantation. While the macronutrient concentrations (P, K, Na, Ca) remained unaffected in both species, the NO_3_^−^ concentration in lettuce decreased under the 3 mg Se L^−1^ treatment, whereas basil showed no significant change. Conversely, Mg content in basil plants increased at the highest dose (3 mg Se L^−1^). Similarly, ref. [85] evaluated the effects of two Na_2_SeO_4_ doses on the nutritional status of two *Valerianella locusta* cultivars (Baron and Gala), revealing differential interactions between Se and macronutrient accumulation, even within the same plant species. At the highest Se level, the Baron cultivar increased its K, P, Ca, Mg, and S leaf contents, whereas the Gala cultivar showed elevated S and P levels under both Se doses (10 μM and 40 μM Se). Contrasting findings have been reported regarding Se’s influence on nitrate (NO_3_^−^) absorption. The study by [86] observed no significant effects of Na_2_SeO_4_ on NO_3_^−^ uptake in spinach, while [87] reported reduced NO_3_^−^ content in green and purple basil, tatsoi, and coriander microgreens under Se application. This discrepancy may stem from the competitive relationship of Se with N assimilation. Since nitrate content in the edible part of leafy vegetables is a significant quality indicator, the European Union has established maximum permissible levels under the Commission Regulation (EU) No 1258/2011 [88]. In our study, the nitrate content of the three cultivated NUS was well below the EU regulatory thresholds, accounting for seasonal growth conditions.

The Se biofortification intervention had a significant impact on the micronutrient status of the three domestic NUS. However, the effects varied considerably among plant species. In dandelion, B content decreased with Se application, whereas in iceplant, 1 μM and 2 μΜ SeO_2_ enhanced B absorption. In contrast, purslane biomass exhibited no change in B content. Fe accumulation in the three NUS was unaffected by Se doses, in contrast with other studies that reported an interaction between Se and Fe [84,87,89], suggesting that Se-nutrient interactions may be context- and species-dependent. SeO_2_ application significantly reduced Mn concentration only in iceplant, which is consistent with the findings of [70] that highlighted negative Se and Mn correlations. However, ref. [74] observed increased Mn content in lettuce following soil application of Se nanomaterials, further underscoring the variability in Se effects, while Zn absorption responded differentially across species. Specifically, in purslane, 1 μM and 2 μM SeO_2_ increased Zn uptake, while the highest dose had an inhibitory effect. Dandelion also exhibited increased an Zn concentration at 2 μM SeO_2_. On the contrary, Zn concentration in iceplant declined progressively with increasing SeO_2_ doses. Scientific literature presents conflicting results regarding Se’s impact on Zn uptake. For instance, ref. [90] reported increased Zn absorption in both roots and leaves of Chinese cabbage when the soil was supplemented with L-Selenomethionine. Conversely, another study [91] indicated that excessive foliar SeO_3_^2−^ (100 and 200 mg L^−1^) reduced Zn content in *Brassica rapa var. rapa* L., whereas the lowest dose (50 mg L^−1^) had no effect [92]. Statistical analysis in the present study demonstrated that the application of 2 μM SeO_2_ enhanced Cu concentration in both purslane and dandelion above-ground biomass. However, the Cu content in iceplant remained unaffected. Existing literature shows considerable heterogeneity in Se’s impact on Cu uptake— in particular, the results of studies reporting Se-induced Cu accumulation in leafy vegetables [74,84]. Conversely, other studies observed reduced Cu uptake when different forms of Se (Na_2_SeO_4_ and SeO_3_^2−^) were applied via NS or foliar application [89,91]. Reference [93] further highlights this variability. In lettuce, fertigation with Na_2_SeO_3_ in nutrient solutions at eight different Se concentrations (0, 5, 10, 20, 40, 60, 80, and 120 µM) increased Cu absorption, reaching its highest levels at 60 μM, beyond which Cu declined. Contrary to this, Na_2_SeO_4_ application reduced Cu content compared to the Se-free control. For all the above findings, it is evident that the interaction between Se and both macro- and microelements are highly context-dependent, and influenced by factors such as Se form, dose, application method, and plant species. While some studies show increased mineral levels, others report reductions. This indicates a complex, genotype-specific interaction rather than a universal trend.

As illustrated in Figure 1, the accumulation of Se enhanced progressively in all three examined NUS with increasing SeO_2_ doses. These findings are consistent with those of numerous studies on Se biofortification across different plant species, Se forms and doses, and cropping systems [68,70,72,73,84,85,86,92,94,95,96,97,98,99,100,101]. Among the three cultivated species, dandelion and iceplant exhibited the highest Se accumulation at 4 μM SeO_2_, with concentrations of 1.1 μg g^−1^ and 0.9 μg g^−1^ Se (fresh biomass), respectively. *Portulaca* exhibited lower Se uptake, with a mean value of 0.6 μg g^−1^ Se (fresh above-ground tissue) at the same dose. These results contrast with a study [95] reporting that aeroponically grown dandelion treated with 7 μg ml^−1^ Se as Na_2_SeO_4_ accumulated less than parsley, lamb’s lettuce, and chicory. This phenomenon, according to the authors, has been ascribed to the dandelion’s underdeveloped root system. For iceplant, ref. [102] found that selenate (SeO_4_^2−^) treatment at 100 μΜ, 300 μΜ, and 400 μM led to high Se accumulation in leaves, suggesting iceplant’s potential as a Se hyperaccumulator. On the other hand, selenite (SeO_3_^2−^) caused significant growth inhibition at 300 μM and total damage at 500 μM. Purslane, a nutrient-rich species, has been widely studied in Se biofortification. Studies have shown that purslane can accumulate Se in various forms (Na_2_SeO_4_ and Na_2_SeO_3_) and through different application methods (foliar application, soil drenching, fertigation) [73,84,100]. The effectiveness of Se biofortification and its dietary impact on consumers is summarised in Table 5. Such indices are analysed in [103]. The corresponding indicators are considered important to include in studies related to biofortification strategies of trace elements, to improve and ensure both efficacy and consumer safety.

It is well documented that, at certain concentrations, selenium is capable of mitigating oxidative stress in plants by stimulating their antioxidant activity [76,104,105]. The findings of the present study reveal species-specific variations in the Se concentrations that positively affect plant antioxidant capacity and secondary metabolite levels. Notably, purslane exhibited the highest antioxidant capacity, total phenolic, and flavonoid content at 1 μM SeO_2_, while iceplant also showed improved antioxidant capacity at the same concentration. In contrast, dandelion achieved its highest antioxidant capacity at elevated SeO_2_ levels. These outcomes are consistent with the respective yield performance of each species. In [105], it is reported that foliar or NS application of Na_2_SeO_4_ at 10 μM enhanced antioxidant activity and total phenolic content in sweet basil, although total flavonoid content remained unaffected by Se doses or application method. Conversely, another study on *Valerianella locusta* L., found that moderate Na_2_SeO_4_ concentrations in the NS increased antioxidant capacity, total phenolic and flavonoid content, whereas higher doses diminished these parameters [68]. These results are consistent with those observed in purslane and iceplant. In addition, elevated Na_2_SeO_3_ concentrations (8 and 10 mg L^−1^) reduced total phenolic and flavonoid content of dandelion seedlings, whereas lower doses have been found to promote these characteristics in comparison to Se-untreated plants [82]. The relationship between Se and the antioxidant properties of plants has been widely documented [72,99,101,103]. Antioxidant activity in plants is essential critical indicator influenced by a complex biochemical interaction within plant extracts [105], although the precise mechanisms of Se’s effects remain incompletely understood. The positive impact of Se, at low concentrations, on the antioxidant properties of plants may be attributed to two key factors: (a) increased non-enzymatic antioxidants such as phenolic compounds, flavonoids, and ascorbic acid [99,104,105], and (b) a correlation between Se and the biosynthesis of glutathione peroxidase (GSx), guaiacol peroxidase (GPOX), thioredoxin reductase (TrxR), and antioxidant enzymes, that play a pivotal role in protecting cells from oxidative damage by scavenging reactive oxygen species (ROS) [82,99,103,104]. However, enzyme responses vary by species and Se dosage. For instance, ref. [106] observed decreased GSH-Px and POD activity at high Se levels, while SOD activity increased progressively. On the other hand, ref. [107] reported elevated SOD, CAT, POD, APX, and GR activity in wild quinoa under increasing Se concentrations. Another study [105] suggested that Se’s influence on antioxidant activity is more likely mediated by its effect on secondary metabolite biosynthesis rather than direct antioxidant action. In conclusion, the decline in antioxidant capacity in purslane and iceplant can be ascribed to the pro-oxidant effect that is evident at elevated concentrations of Se [108,109]. An alternative hypothesis, that requires further examination, is that excessive Se may upregulate antioxidant enzyme activities (POD, SOD, and GSx), while suppressing secondary metabolite pathways, explaining the reduced antioxidant properties observed in purslane and iceplant under excess Se.

## 4. Materials and Methods

### 4.1. Plant Material, Growth Conditions, and Cultivation Period

The experiment was conducted in the greenhouse facilities of the Agricultural University of Athens (AUA), (37.98224806153772, 23.704695255748245), utilising the equipment of AUA’s Vegetable Production Laboratory to assess plant nutrient status. Seedlings were prepared using commercial seeds (*Portulaca oleracea* L., *Taraxacum officinale* L., and *Mesembryanthemum crystallinum* L.) from Fitotech Industrial and Commercial S.A, Athens Greece. Fully developed seedlings were transplanted at the growing stage of four true leaves. Table 6 shows the dates of sowing, transplanting, and harvesting. Cultivation was carried out in open soilless system, using bags of expanded perlite, with a capacity of five plants per sack. Prior to transplantation, all bags were pre-soaked with a starter nutrient solution (NS). After drainage through slits at the bottom of the sacks, irrigation commenced with a vegetative-stage NS. The NS was formulated based on plant nutritional requirements and international standards, using the Nutrisense Decision Support System (https://nutrisense.online/, accessed on 1 April 2025) [110]. The composition of the vegetative NS was the same for the three plant species and it is presented in Table 7. In order to achieve uniform crop irrigation, a constant supply of drips at a rate of 2 L per hour was utilized. Finally, the drainage fraction was maintained at approximately 30% of the total NS supplied through daily measurements of the excess NS. At the same time, the pH and EC of the runoff NS were measured simultaneously to control rhizosphere conditions.

### 4.2. Experiment Design

The experiment was designed following the fundamental principles of agricultural experimentation, namely randomisation, repetition, and local control. To ensure homogeneity across treatments and uniform plant growth conditions, a completely randomized design model was employed. The experiment involved the administration of SeO_2_ (Sigma-Aldrich, St. Louis, MO, USA) to the plants, according to a previous study [72], at four distinct concentrations: 0 μM (control), 1 μM, 2 μM, and 4 μM. More specifically, a 1 mM Se stock solution was initially prepared, followed by dilution to attain the appropriate concentrations. Each treatment was replicated four times per plant species, resulting in a total of 80 plants per species (four treatments × four replicates × five plants per experimental unit).

### 4.3. Plant Growth

The harvesting of the plants was conducted prior to the flowering stage. Total plant yield was determined by harvesting samples from each treatment and measuring their fresh weight (FW). Additionally, leaf area and leaf number were recorded for each sample. Following the harvest, samples were placed in a forced-air drying oven at 60 °C for 10 d to achieve complete desiccation. After drying, dry weight (DW) and dry matter content (DMC) were measured. This step ensured the removal of all moisture, preparing the samples for subsequent pulverization, and dry ashing [111].

### 4.4. Mineral Concentration

The mineralisation of the dry plant tissue samples was performed in accordance with the dry ashing protocol, utilising a combustion chamber manufactured by Lion High Therm. A quantity of 0.5 g of each sample was placed within porcelain capsules, which were then subjected to a combustion process at a temperature of 500 °C for a duration of 8 h. The measurement of the concentration of some macronutrients and trace elements (Ca^2+^, Mg^2+^, Fe^2+^, Mn^2+^, Zn^2+^, Cu^2+^) was conducted utilizing an atomic absorption spectrophotometer (AA-7000, Kyoto, Japan), and the determination of P, BO_3_^−^, NO_3_^−^, concentration was conducted colorimetrically using a spectrophotometer (Biochrom Ltd. Anthos Zenyth 200rt Microplate reader, Cambridge, UK), while Na^+^ and K^+^ concentrations were measured by the method of atomic emission spectroscopy (AES) using a Sherwood Flame Photometer 410 (Cambridge, UK). The determination of total nitrogen (total Kjeldahl nitrogen) was carried out according to the process of wet digestion, specifically with the Kjeldahl method [112]. Finally, the Se concentration in plant tissue was determined in the laboratory facilities of the Department of Agricultural, Food and Forestry Sciences of the University of Palermo, using an ICP-MS (Plasma Quant MS Elite, Jena, Germany) [72].

### 4.5. Dietary Se Intake and Health Risk Indicators of Biofortified NUS

Based on a previous study [103], the Se estimated daily intake (EDI) index was determined according to the concept of a 50 g daily consumption of biofortified plants. Moreover, the EDI % was expressed as percentage of the Se Recommended Dietary Allowance (RDA) recommended by the responsible authorities worldwide [30,32]. Finally, in order to evaluate the potential health implications associated with the consumption of biofortified NUS, the health risk index (HRI) was determined as the ratio between the estimated daily intake (EDI) and the tolerable upper intake level.

### 4.6. Determination of Plant Biochemical Profile

The overall antioxidant activity of the tested NUS was crucial to ascertain, and in order to determine the impact of the Se on the biochemical profile of the plants, it was essential to freeze-dry the fresh plant tissue samples and subject them to methanolic extraction, as described in [113]. The Trolox Equivalent Antioxidant Capacity (TEAC) assay and the Ferric Reducing Antioxidant Power (FRAP) assay were conducted, with the values expressed in Trolox equivalent per g of dry biomass [114,115]. The total phenolic content (TPC) was determined following the Folin–Ciocalteu (FC) method [116], while the total flavonoid content (TFC) of the plant samples was determined by means of the aluminium chloride colorimetric method [117].

### 4.7. Statistical Analysis

The statistical analysis was conducted utilising the STATISTICA 12.0 software package, (StatSoft Inc., Tulsa, OK, USA). In order to assess data normality, the Shapiro–Wilk test was used. The data were subjected to one-way and two-way ANOVA, and the differences among the mean values for each treatment were tested using Duncan’s multiple range test. A *p* value less than 0.05 was considered to be statistically significant. The data are presented in graphs as means ± standard error of the mean of four replicates. The graphs were generated using GraphPad Prism 8.

## 5. Conclusions

This study demonstrates that SeO_2_ is as an effective selenium form for biofortification when incorporated into nutrient solutions, particularly for underutilised plant species with inherent nutritional value and environmental resilience. Our results confirm that SeO_2_ application via the rootzone can effectively enhance growth characteristics, with purslane showing increased biomass and leaf area across all tested doses, dandelion responding best to higher concentrations, and iceplant exhibiting sensitivity at the highest dose (4 μM). All species accumulated Se in a dose-dependent manner, but only iceplant showed clear growth inhibition at higher doses. The impact of Se on nutrient uptake was complex and species-specific, affecting macronutrients like nitrogen and phosphorus as well as micronutrients such as zinc, copper, and manganese. At optimal doses, it enhanced antioxidant compounds and enzymes, but excessive Se acted as a pro-oxidant. For commercial adoption, the recommended SeO_2_ levels are 1 μM for purslane, 4 μM for dandelion, and 1 μM for iceplant. However, the integration of SeO_2_ into an open hydroponic system can result in the generation of selenium-rich nutrient runoff, thereby posing a risk of environmental contamination, particularly to aquatic ecosystems, if effluent is not adequately contained and treated. In summary, this study underscores the potential of selenium biofortification to enhance the growth and nutritional quality of underutilised plant species. However, successful implementation requires careful consideration of species-specific tolerance levels, Se formulation, and application methods to maximize benefits while avoiding toxicity. Future research should further explore the molecular mechanisms behind Se-induced stress responses and nutrient interactions to optimize biofortification strategies for diverse crops.

## Figures and Tables

**Table 1:** Impact of different SeO_2_ treatments on the yield, number of leaves, leaf area, shoot dry weight, and above-ground biomass dry matter content of hydroponically grown purslane, dandelion and iceplant.

Plant Species	Se Treatment	Yield	Leaf Number	Leaf Area	Dry Weight	Dry Matter Content
	(SeO_2_ μM)	(g plant^−1^)	(n. plant^−1^)	(cm plant^−1^)	(g plant^−1^)	(%)
*P. oleracea* L.	0	96.7 b	267.3 b	806.5 b	14.0	4.7 ab
	1	119.0 a	356.3 a	1067.0 a	16.3	4.5 b
	2	112.3 a	316.0 ab	948.7 ab	15.2	4.7 ab
	4	112.6 a	382.9 a	1024.3 a	17.4	4.8 a
Statistical Significance		**	*	*	NS	*
*T. officinale* L.	0	14.4 b	19.9 b	374.9 c	1.9 b	13.3 a
	1	14.6 b	19.6 b	376.9 c	1.9 b	13.0 b
	2	15.4 b	23.3 a	450.6 b	2.0 b	12.9 c
	4	19.2 a	23.8 a	560.6 a	2.4 a	12.4 d
Statistical Significance		***	**	***	***	***
*M. crystallinum* L.	0	97.7 b	86.6 bc	749.4 b	2.3	2.7 a
	1	118.9 a	110.7 a	994.8 a	2.7	2.4 b
	2	114.5 a	104.0 ab	982.0 a	2.6	2.5 b
	4	88.2 b	80.3 c	721.2 b	2.3	2.8 a
Statistical Significance		**	*	**	NS	**

Mean values within the same column followed by different letters indicate statistically significant differences according to Duncan’s multiple range test at *p* < 0.05. *, **, and *** denote significance at *p* < 0.05, *p* < 0.01 and *p* < 0.001, respectively. NS indicates non-significance.

**Table 2:** Impact of different SeO_2_ doses on macronutrient [total nitrogen (TN), P, K, Ca, Mg, and Na] concentrations in dry and nitrate–nitrogen (NO_3_-N) in the fresh plant tissues of the examined NUS.

Plant Species	Se Treatment	TN	NO_3_-N	P	K	Ca	Mg	Na
	(SeO_2_ μM)	%	(mg kg^−1^ FW)	(mg g^−1^)	(mg g^−1^)	(mg g^−1^)	(mg g^−1^)	(mg g^−1^)
*P. oleracea* L.	0	5.5	1397.1	6.9	49.0	21.4	9.4	6.0
	1	5.8	1363.7	6.4	47.8	23.1	9.0	6.9
	2	5.8	1482.1	6.8	50.0	20.6	8.5	7.0
	4	5.5	1483.0	6.9	49.0	24.2	8.9	6.4
Statistical Significance		NS	NS	NS	NS	NS	NS	NS
*T. officinale* L.	0	4.2 b	572.0 b	6.7 a	23.5	5.0	3.9	0.3
	1	4.1 b	626.8 b	5.9 ab	25.5	5.2	3.8	0.2
	2	4.4 ab	873.1 a	5.4 b	25.3	5.2	4.0	0.3
	4	4.6 a	872.5 a	5.2 b	23.8	5.7	4.3	0.3
Statistical Significance		*	***	*	NS	NS	NS	NS
*M. crystallinum* L.	0	5.0 a	707.3 a	4.9 a	48.3	4.5 a	6.2 a	15.0 b
	1	4.7 b	498.7 b	4.9 a	52.3	4.4 a	5.8 a	15.0 b
	2	4.4 c	468.6 b	4.2 b	50.0	3.8 a	4.8 b	16.5 b
	4	3.8 d	449.0 b	3.6 c	53.8	2.6 b	3.1 c	20.0 a
Statistical Significance		***	***	***	NS	**	***	**

Mean values within the same column followed by different letters indicate statistically significant differences according to Duncan’s multiple range test at *p* < 0.05. *, **, and *** denote significance at *p* < 0.05, *p* < 0.01, and *p* < 0.001, respectively. NS indicates non-significance.

**Table 3:** Impacts of different SeO_2_ doses on micronutrient (B, Fe, Mn, Zn, and Cu) concentrations in the dry plant tissues of the examined NUS.

Plant Species	Se Treatment	B	Fe	Mn	Zn	Cu
	(SeO_2_ μM)	(μg g^−1^)	(μg g^−1^)	(μg g^−1^)	(μg g^−1^)	(μg g^−1^)
*P. oleracea* L.	0	37.9	14.6	42.1	64.0 b	6.7 c
	1	41.5	17.3	44.7	76.8 a	7.7 c
	2	38.2	17.6	47.4	82.0 a	13.0 a
	4	47.7	19.5	42.6	64.5 b	11.2 b
Statistical Significance		NS	NS	NS	**	***
*T. officinale* L.	0	98.7 a	77.8	92.1	45.4 b	9.7 b
	1	79.5 b	77.1	92.6	48.6 b	9.5 b
	2	74.9 b	82.4	97.9	58.7 a	13.5 a
	4	71.3 b	82.4	97.2	45.0 b	13.3 a
Statistical Significance		**	NS	NS	*	***
*M. crystallinum* L.	0	19.6 c	25.0	81.9 a	67.2 a	14.2
	1	29.4 b	24.4	48.9 b	29.3 b	11.7
	2	53.7 a	24.2	52.3 b	15.2 bc	12.7
	4	24.6 bc	26.9	52.1 b	11.4 c	10.3
Statistical Significance		***	NS	**	***	NS

Mean values within the same column followed by different letters indicate statistically significant differences according to Duncan’s multiple range test at *p* < 0.05. *, **, and *** denote significance at *p* < 0.05, *p* < 0.01, and *p* < 0.001, respectively. NS indicates non-significance.

**Table 4:** Impacts of different SeO_2_ doses and plant species on Se concentration in fresh plant tissues of the examined NUS.

Se Treatment	Plant Species	Se
(SeO_2_ μM)		μg g^−1^ FW
Main effects (Plant Species)		
*P. oleracea* L.		0.3 c
*T. officinale* L.		0.5 a
*M. crystallinum* L.		0.4 b
Main effects (Se Treatment)		
	0	0.1 d
	1	0.3 c
	2	0.5 b
	4	0.9 a
Statistical Significance		
Plant Species (PS)		***
Se Treatment (Se)		***
PS × Se		***

Mean values within the same column followed by different letters indicate statistically significant differences according to Duncan’s multiple range test at *p* < 0.05. *** denotes significance at *p* < 0.001.

**Table 5:** Impacts of different SeO_2_ doses and plant species on Se estimated daily intake (EDI), Se estimated dietary intake (EDI %), and health risk index (HRI) of Se intake.

Plant Species	Se Treatment	EDI	EDI	HRI
	(SeO_2_ μM)	(g day^−1^)	(%)	
*P. oleracea* L.	0	2.4 d	3.4 d	0.01 d
	1	9.7 d	13.9 d	0.02 d
	2	18.3 c	26.1 c	0.05 c
	4	29.7 b	42.4 b	0.07 b
*T. officinale* L.	0	4.4 d	6.3 d	0.01 d
	1	18.5 c	26.5 c	0.05 c
	2	33.3 b	47.5 b	0.08 b
	4	53.0 a	75.8 a	0.13 a
*M. crystallinum* L.	0	2.6 d	3.7 d	0.01 d
	1	9.7 d	13.9 d	0.02 d
	2	20.8 c	29.7 c	0.05 c
	4	47.0 a	67.1 a	0.12 a
Main Effect				
*P. oleracea* L.		15.0 c	21.5 a	0.04
*T. officinale* L.		27.3 a	39.0 c	0.07
*M. crystallinum* L.		20.0 b	28.6 b	0.05
	0	3.1 d	4.5 d	0.01 d
	1	12.7 c	18.1 c	0.03 c
	2	24.1 b	34.5 b	0.06 b
	4	43.2 a	61.8 a	0.11 a
Statistical Significance				
Plant Species (PS)		***	***	***
Se Concentration (Se)		***	***	***
PS × Se		***	***	***

Mean values within the same column followed by different letters indicate statistically significant differences according to Duncan’s multiple range test at *p* < 0.05. *** denotes significance at *p* < 0.001.

**Table 6:** Sowing, transplanting and harvesting dates.

Plant Species	Sowing	Transplanting	Harvesting
*Portulaca oleracea* L.	10 June	6 July	25 July
*Taraxacum officinale* L.	12 April	8 June	26 July
*Mesembryanthemum crystallinum* L.	14 April	8 June	6 July

**Table 7:** Chemical composition of starter and vegetative nutrient solution (NS).

Nutrient	Starter	Vegetative	Unit
EC	2.4	2.4	dS/m
pH	5.6	5.6	
NO^3−^	13.08	14.00	mM
K^+^	8.09	7.00	mM
Ca^2+^	4.77	5.00	mM
Mg^2+^	3.10	2.00	mM
SO_4_^2−^	4.53	3.06	mM
H_2_PO_4_^−^	1.40	1.50	mM
NH_4_^+^	1.11	0.82	mM
Fe	20.00	30.00	μM
Mn^2+^	9.00	10.00	μM
Zn^2+^	5.00	7.00	μM
B	30.00	35.00	μM
Cu^2+^	0.80	0.80	μM
Mo	0.50	0.80	μΜ
Cl^−^	0.40	0.40	μΜ
K/(K + Ca + Mg)	0.51	0.50	mol/mol
Ca/(K + Ca + Mg)	0.30	0.36	mol/mol
Mg(K + Ca + Mg)	0.19	0.14	mol/mol
N/K	1.75	2.12	mol/mol
NH_4_-N/Total-N	0.08	0.06	mol/mol

## Data Availability

The original contributions presented in this study are included in the article.
